# Supplementary figures and images for: Comparative mitogenomic analysis provides evolutionary insights into Formica (Hymenoptera: Formicidae)
Source: PLoS One. 2024 Jun 10;19(6):e0302371. doi: 10.1371/journal.pone.0302371 (PMC11164359; doi:10.1371/journal.pone.0302371)

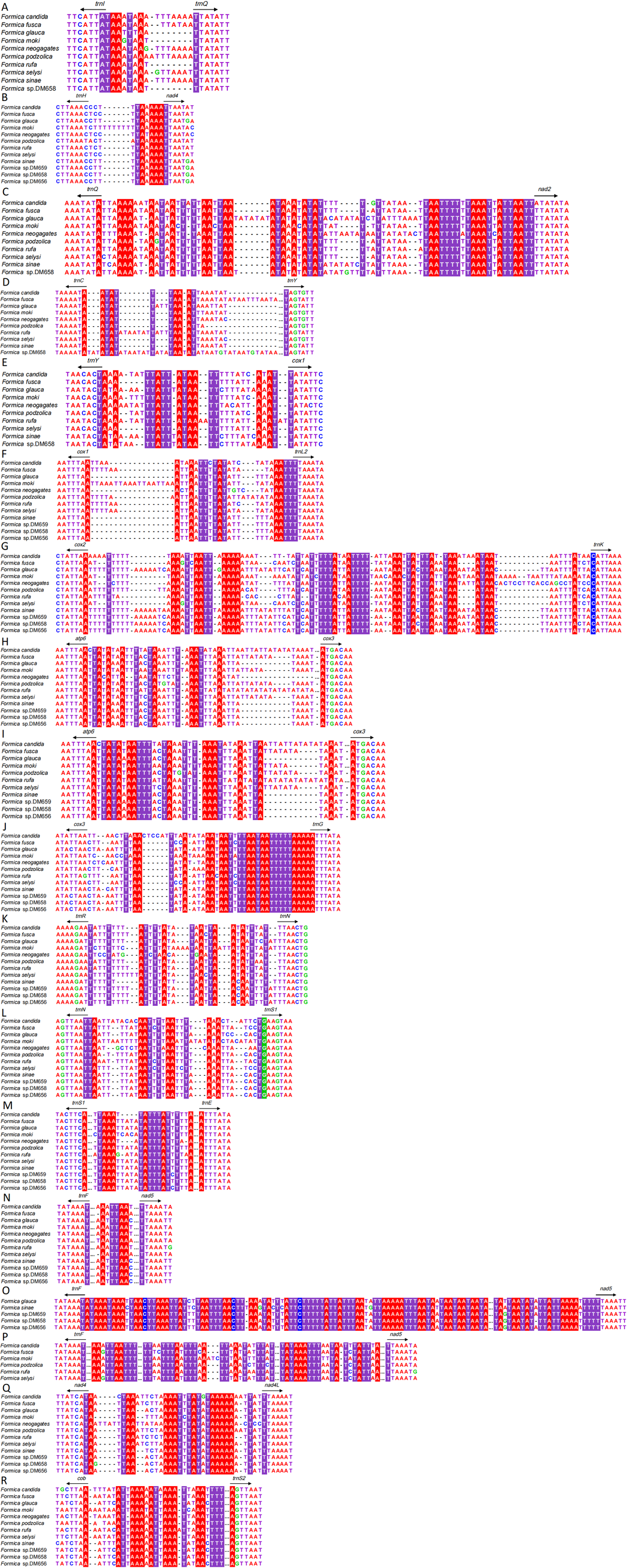

Supplement: S2 Fig — (A) The intergenic spacer between trnI and trnQ. (B) The intergenic spacer between trnH and nad4. (C) The intergenic spacer between trnQ and nad2. (D) The intergenic spacer between trnC and trnY. (E) The intergenic spacer between trnY and cox1. (F) The intergenic spacer between cox1 and trnL2. (G) The intergenic spacer between cox2 and trnK. (H) The intergenic spacer between atp6 and cox3. (I) The intergenic spacer between atp6 and cox3, except in F. neogagates. (J) The intergenic spacer between cox3 and trnG. (K) The intergenic spacer between trnR and trnN. (L) The intergenic spacer between trnN and trnS1. (M) The intergenic spacer between trnS1 and trnE. (N) The intergenic spacer between trnF and nad5. (O) The intergenic spacer between trnF and nad5 in F. glauca, F. sinae, Formica sp.DM659, Formica sp.DM658, and Formica sp.DM656. (P) The intergenic spacer between trnF and nad5 in F. candida, F. fusca, F. moki, F. podzolica, F. rufa, and F. selysi. (Q) The intergenic spacer between nad4 and nad4L. (R) The intergenic spacer between cob and trnS2. (TIF) [file pone.0302371.s002.tif]

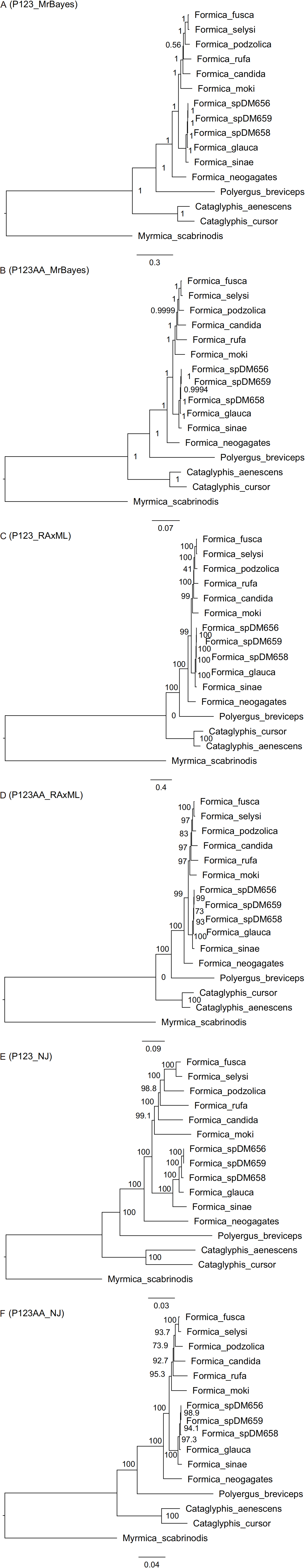

Supplement: S3 Fig — (TIF) [file pone.0302371.s003.tif]
