# Supplementary material for: Comparative mitogenomic analysis provides evolutionary insights into Formica (Hymenoptera: Formicidae)
Source: PLoS One. 2024 Jun 10;19(6):e0302371. doi: 10.1371/journal.pone.0302371 (PMC11164359; doi:10.1371/journal.pone.0302371)
Supplement: S1 Table — (DOCX) [file pone.0302371.s004.docx]

Table S1. Sampling information of the two *Formica* species that were newly sequenced in this study.

| Family | Species | Sampling site | Voucher specimen | Altitude | Coordinate |
| --- | --- | --- | --- | --- | --- |
| Formicidae | *Formica candida* | Qumalai County, Qinghai Province, China | CX-Fa | 4500 m | 95°50′E, 34°07'N |
| Formicidae | *Formica glauca* | Altay City, Xinjiang Uygur Autonomous Region, China | ALTFg | 500 m | 87°33′E, 47°42'N |
