# Supplementary material for: Comparative mitogenomic analysis provides evolutionary insights into Formica (Hymenoptera: Formicidae)
Source: PLoS One. 2024 Jun 10;19(6):e0302371. doi: 10.1371/journal.pone.0302371 (PMC11164359; doi:10.1371/journal.pone.0302371)
Supplement: S2 Table — Species that were newly sequenced in this study are labelled with an asterisk. (DOCX) [file pone.0302371.s005.docx]

Table S2. Characteristics of mitogenomes of 16 Formicidae species used in this study. Species that were newly sequenced in this study are highlighted with an asterisk.

| Family | Subfamliy | Species | GenBank accession numbers | Genome Size (bp) |
| --- | --- | --- | --- | --- |
| Formicidae | Formicinae | *Cataglyphis aenescens* | NC_060872 | 17197 |
| Formicidae | Formicinae | *Cataglyphis cursor* | BK012526 | 17439 |
| Formicidae | Formicinae | *Formica candida** | ON408245 | 16565 |
| Formicidae | Formicinae | *Formica fusca* | NC_026132 | 16673 |
| Formicidae | Formicinae | *Formica glauca** | ON408246 | 16492 |
| Formicidae | Formicinae | *Formica moki* | BK012606 | 16932 |
| Formicidae | Formicinae | *Formica neogagates* | BK012298 | 17431 |
| Formicidae | Formicinae | *Formica podzolica* | BK012706 | 14190 |
| Formicidae | Formicinae | *Formica rufa* | MT862420 | 14184 |
| Formicidae | Formicinae | *Formica selysi* | NC_026711 | 16752 |
| Formicidae | Formicinae | *Formica sinae* | NC_060873 | 17432 |
| Formicidae | Formicinae | *Polyergus brevicep* | BK012290 | 14615 |
| Formicidae | Formicinae | *Formica* sp.DM656 | MT941914 | 12064 |
| Formicidae | Formicinae | *Formica* sp.DM659 | MT941917 | 12626 |
| Formicidae | Formicinae | *Formica* sp.DM658 | MT941916 | 14348 |
| Formicidae | Myrmicinae | *Myrmica scabrinodis* | NC_026133 | 15310 |
