# Supplementary material for: Comparative mitogenomic analysis provides evolutionary insights into Formica (Hymenoptera: Formicidae)
Source: PLoS One. 2024 Jun 10;19(6):e0302371. doi: 10.1371/journal.pone.0302371 (PMC11164359; doi:10.1371/journal.pone.0302371)
Supplement: S3 Table — (DOCX) [file pone.0302371.s006.docx]

Table S3. Saturation test for each of the 13 protein coding genes (PCG) sequences, concentration of 13 PCG sequences, and three positions of 13 PCGs as implemented in DAMBE. (Iss: index of substitution saturation; Iss.cS: the critical Iss value)

| Dataset | Iss | Iss.cS | *P* |
| --- | --- | --- | --- |
| *atp6* | 0.1921 | 0.7377 | < 0.01 |
| *atp8* | 0.491 | 0.6383 | < 0.01 |
| *cob* | 0.2092 | 0.7701 | < 0.01 |
| *cox1* | 0.1779 | 0.7932 | < 0.01 |
| *cox2* | 0.211 | 0.7478 | < 0.01 |
| *cox3* | 0.1841 | 0.7495 | < 0.01 |
| *nad1* | 0.2034 | 0.7691 | < 0.01 |
| *nad2* | 0.4112 | 0.7653 | < 0.01 |
| *nad3* | 0.219 | 0.6866 | < 0.01 |
| *nad4* | 0.2174 | 0.7798 | < 0.01 |
| *nad4L* | 0.1944 | 0.6732 | < 0.01 |
| *nad5* | 0.2275 | 0.79 | < 0.01 |
| *nad6* | 0.2537 | 0.7225 | < 0.01 |
| 13P123 | 0.2365 | 0.8502 | < 0.01 |
| Position 1st | 0.1882 | 0.827 | < 0.01 |
| Position 2nd | 0.1189 | 0.827 | < 0.01 |
| Position 3rd | 0.4551 | 0.827 | < 0.01 |
