# Supplementary material for: Comparative mitogenomic analysis provides evolutionary insights into Formica (Hymenoptera: Formicidae)
Source: PLoS One. 2024 Jun 10;19(6):e0302371. doi: 10.1371/journal.pone.0302371 (PMC11164359; doi:10.1371/journal.pone.0302371)
Supplement: S4 Table — (DOCX) [file pone.0302371.s007.docx]

Table S4. The best partitioning schemes and substitution models selected by IQ-TREE for the two datasets.

| Dataset | Subset | Best-fit scheme | Model |
| --- | --- | --- | --- |
| P123 | P1 | *atp6*_pos1, *cob*_pos1, *cox1*_pos1, *cox2*_pos1, *cox3*_pos1 | TIM2+F+I |
|  | P2 | *atp6*_pos2, *atp8*_pos2, *cob*_pos2, *cox1*_pos2, *cox2*_pos2, *cox3*_pos2, *nad1*_pos2, *nad2*_pos2, *nad3*_pos2, *nad4*_pos2, *nad4L*_pos2, *nad5*_pos2, *nad6*_pos2 | K3Pu+F+I+G4 |
|  | P3 | *atp6*_pos3, *atp8*_pos3, *cob*_pos3, *cox1*_pos3, *cox2*_pos3, *cox3*_pos3, *nad2*_pos3, *nad3*_pos3, *nad6*_pos3 | TIM+F+I+G4 |
|  | P4 | *atp8*_pos1, *nad1*_pos1, *nad2*_pos1, *nad3*_pos1, *nad4*_pos1, *nad4L*_pos1, *nad5*_pos1, *nad6*_pos1 | TIM3+F+I+G4 |
|  | P5 | *nad1*_pos3, *nad4*_pos3, *nad4L*_pos3, *nad5*_pos3 | TIM+F+G4 |
| 13P123AA | P1 | *atp6*, *atp8*, *cob*, *cox2*, *cox3*, *nad1*, *nad2*, *nad3*, *nad4*, *nad4L*, *nad5*, *nad6* | mtMAM+F+G4 |
|  | P2 | *cox1* | mtART+G4: |
