# Supplementary material for: Comparative mitogenomic analysis provides evolutionary insights into Formica (Hymenoptera: Formicidae)
Source: PLoS One. 2024 Jun 10;19(6):e0302371. doi: 10.1371/journal.pone.0302371 (PMC11164359; doi:10.1371/journal.pone.0302371)
Supplement: Attachment — Submitted filename: Comments to PONE-D-23-17435.docx [file pone.0302371.s009.docx]

1. To ensure the accuracy of mitochondrial genome, there should be sample duplication during sequencing. Whether there is sample duplication is not mentioned in this manuscript, please indicate if there is sample duplication.

2. There are many cases of repeated descriptions in the manuscript, such as excessive description of Spacer1-14 of IGSs in 3.3, which can be deleted according to their importance and uniqueness to make the manuscript language concise.

3. There are repetitions of the results in the discussion, and there are also incorrect statements. For example, in the discussion of phylogenetic results of group 1 and group 2, there is a description of the genetic relationship of five species in group 2, which should belong to group 1, but it is in group 2, which needs to be carefully verified.

4. There are missing words in the manuscript, which need to be checked the whole text. For example, the calculation method of AT-skew and GC-skew values in 2.3 is omitted, and "negative" is missing in "Ka/Ks<1 indicating selection" in 4.3, which should be "Ka/Ks<1 indicating negative selection", and so on.

5. AA datasets of insect mitochondrial genomes was usually applied to reconstruct the phylogeny in various taxa levels of insects. Author found “there were a few different relationships within *Formica* based on different datasets”, but all support the relationship among three genus. What is the reason? That should be discussed.

6. The literature should be added in the end of follow sentence “This unstable phylogenetic relationships of *Formica* were also reported in previous studies ” in 4.5.
