# Supplementary material for: Comparative mitogenomic analysis provides evolutionary insights into Formica (Hymenoptera: Formicidae)
Source: PLoS One. 2024 Jun 10;19(6):e0302371. doi: 10.1371/journal.pone.0302371 (PMC11164359; doi:10.1371/journal.pone.0302371)
Supplement: Attachment — Submitted filename: Reply to Reviews Comments-Submission.docx [file pone.0302371.s010.docx]

**For Reviewer #1**

The manuscript investigated two mt-genomes of the genus Formica and resconstructed the phylogenic relationship within the genus using those datasets. The results will be a potential reference for the understanding of insects mt-genome and their applification for the phlogeny within genus.

**Response**: We thank the reviewer’s positive comments on the manuscript.

**For Reviewer #2**

1. I suggest the authors marked the ‘Line numbers’ throughout the manuscript, it would be easier to track the comments.

**Response**: We have provided “Line numbers” throughout the revised manuscript, as suggested.

2. In the section ‘3.2. Nucleotide composition and codon usage’, keep the number ‘3274’ and ‘3712’ (be 3,712) the same style with previous content, please check with all the manuscript.

**Response**: We have revised the manuscript, as suggested.

3. In the section ‘3.3. Intergenic spacers’, I suggest rephrase the intergenic spacers (IGSs), especially you don’t need to list all the 14 spacers in details, please emphasize most important results.

**Response**: We have rephrased the intergenic spacers, by deleting some unnecessary descriptions of 14 IGSs to emphasize the most important results, as suggested.

4. In the section ‘3.6. Mitochondrial phylogeny of Formica’, there is a typo error ‘group 2 including five species, with.’

**Response**: We have changing “group 2 including five species, with” into “group 2 including five species”, as suggested.

5. In the section ‘4.5. Formica Phylogeny and intergenic spacers’, the authors said ‘unstable phylogenetic relationships of Formica’, which phylogeny gives most confident their relationships? What’s the explanation?

**Response**: We thank the reviewer’s invaluable comments. The *Formica* phylogeny (Figure 9A) obtained from the P123 dataset may be most confident, as reported by Goropashnaya et al. (PLoS One, 2012). We discussed the unstable phylogenetic relationships of *Formica* in the Discussion section.

6. Figure 9, I suggest the authors label all the nodes with bootstrap values.

**Response**: We thank the reviewer’s invaluable comments. Given the six *Formica* phylogenies have almost consistent phylogenetic relationships and most support values were more than 90 and 0.9, we integrated all phylogenetic results (Figure 3A) into a common phylogenetic backbone and used black dots to denote support values greater than 90 (RA x ML) and 0.9 (MrBayes and NJ), which did not affect the understanding of the phylogenies. We also provided all phylogenetic trees with specific support values for each node in Figure 3A.
